# Rehabilitative efficacy of cognitive behavioral therapy-based intervention for chronic fatigue syndrome: A systematic review and meta-analysis

**DOI:** 10.1097/MD.0000000000050765

**Published:** 2026-10-09

**Authors:** Xiaoyu Jiang, Junnan Liu, Shulun Li, Qian Pei, Qiang Huang

**Affiliations:** a Department of Rehabilitation Medicine, Beijing Jishuitan Hospital, Capital Medical University, No. 31, Xinjiekou East Street, Xicheng District, Beijing, China.

**Keywords:** chronic fatigue syndrome, cognitive behavioral therapy, long-term efficacy, meta-analysis, psychological health

## Abstract

**Background:**

Cognitive behavioral therapy (CBT) has been used for chronic fatigue syndrome (CFS), but previous studies have shown inconsistencies and divergent long-term outcomes. This study aims to assess the effectiveness of CBT in improving fatigue, physical functioning, and psychological health in patients with CFS.

**Methods:**

We searched PubMed, Web of Science, Embase, and the Cochrane Library from their inception to March 2025. Studies designed as randomized controlled trials, enrolling patients diagnosed with CFS who received CBT as a treatment intervention delivered face-to-face, in groups, or online were included. The primary outcome was the fatigue score, and the secondary outcomes were the physical function score, psychological outcomes, and adverse events. The quality of the included studies was evaluated using the Cochrane Assessment Tool for Risk of Bias Version 2 (RoB 2; The Cochrane Collaboration). Heterogeneity was detected by subgroup analysis and sensitivity analysis.

**Results:**

Fifteen randomized controlled trials with 2270 participants were involved. Meta-analysis demonstrated that CBT yielded significant posttreatment improvements across key outcomes. Fatigue severity was notably reduced, as measured by the Chalder Fatigue Questionnaire (standardized mean difference [SMD] = −0.44, 95% confidence interval [CI]: −0.57–−0.32) and the Checklist Individual Strength (SMD = −0.88, 95% CI: −1.16–−0.60). Physical functioning (physical function subscale of the Short Form Health Survey-36) subscale also improved significantly (SMD = 0.32, 95% CI: 0.23–0.41). Psychological health benefits were evident through reduced Symptom Checklist 90 (SMD = −0.44, 95% CI: −0.62–−0.26) and specific decreases in anxiety (Hospital Anxiety and Depression Scale–anxiety scale: SMD = −0.28, 95% CI: −0.46–−0.10) and depression (Hospital Anxiety and Depression Scale–depression scale: SMD = −0.27, 95% CI: −0.45–−0.09). Follow-up analyses confirmed sustained benefits: short-term (≤ 6 months) showed continued fatigue reduction (Chalder Fatigue Questionnaire: SMD = −0.29, 95% CI: −0.55–−0.03) and improved physical function (physical function subscale of the Short Form Health Survey-36: SMD = 0.31, 95% CI: 0.03–0.58); long-term (6–24 months) maintained fatigue relief (SMD = −0.35, 95% CI: −0.50–−0.19) and functional gains (SMD = 0.21, 95% CI: 0.06–0.37). Safety analysis showed no statistically significant difference in adverse event rates (odds ratio = 0.61, 95% CI: 0.36–1.01, *P* = .060).

**Conclusion:**

Based on our results, CBT may be an effective option for CFS, but further research is warranted.

## 1. Introduction

Chronic fatigue syndrome (CFS), also known as myalgic encephalomyelitis,^[1]^ is a medically unexplained illness characterized by severe fatigue limiting normal daily activities for at least 6 months, accompanied by problems with cognitive dysfunction,^[2]^ unrefreshing sleep,^[3]^ exacerbation of symptoms following physical or mental efforts (postexertional malaise [PEM]),^[4]^ and widespread musculoskeletal pain.^[5]^ Current research predominantly suggests CFS arises from multifactorial influences,^[6]^ namely the interaction of genetic susceptibility,^[7]^ environmental factors (such as viral infections),^[8,9]^ and psychological factors,^[10]^ potentially leading to dysregulation of immune,^[11]^ neurological, and energy metabolism processes.^[12]^

Estimates of global CFS prevalence vary considerably, primarily due to differing case-definition methodologies. For instance, a prevalence of 8.1% reported in European primary care studies^[13]^ contrasts sharply with rates as low as 0.89% estimated using stricter criteria such as those of the CDC.^[14]^ This discrepancy reveals a fundamental diagnostic conundrum rather than genuine epidemiological variation. The absence of standardized diagnostic biomarkers and the use of heterogeneous criteria complicate accurate disease burden assessment and cross-study comparisons, underscoring the necessity for critical synthesis of existing evidence.

The recent novel coronavirus pandemic has brought renewed urgency and perspective to CFS research through the emergence of “long corona virus disease (COVID)” (post-COVID condition).^[15]^ Significant parallels exist between the 2 in core symptoms^[16]^ such as debilitating fatigue, cognitive impairment, and PEM.^[17]^ Crucially, emerging research suggests they may share pathophysiological pathways, including persistent immune dysregulation^[18]^ and autonomic dysfunction.^[19]^ This convergent mechanism not only enhances the biological plausibility of CFS but also positions CFS-related management strategies as potential interventions for long COVID.^[20,21]^

The management of CFS remains challenging, with no cure currently available.^[4]^ Treatment approaches encompass both pharmacological and non-pharmacological interventions. While medication may alleviate symptoms in myalgic encephalomyelitis/CFS patients^[5]^ (such as pain and sleep disturbances) and address comorbidities like postural orthostatic tachycardia syndrome, antidepressant treatments like long-term methylphenidate only benefit some CFS patients with attention difficulties,^[22]^ while stimulants such as methylphenidate or amphetamines may exacerbate PEM.^[23]^ Some clinical studies also report that supplements such as coenzyme Q10 combined with reduced nicotinamide adenine dinucleotide may reduce cognitive fatigue and improve health-related quality of life in CFS patients.^[24,25]^ However, the current evidence base for pharmacological treatments is generally limited, and risks must be carefully weighed against benefits. In contrast, non-pharmacological interventions, notably cognitive behavioral therapy (CBT) and graded exercise therapy, receive greater recommendation in clinical guidelines.^[4,5]^

CBT is a psychotherapy based on the premise that individuals’ symptoms, emotions, cognitive functions, and behaviors are interconnected, and that interventions targeting thoughts or behaviors might improve emotional distress and alleviate symptoms of certain diseases.^[26,27]^ Several studies have demonstrated that CBT could effectively improve a range of psychological and physical symptoms (e.g., fatigue, anxiety, and depression) in patients with CFS following short-term interventions.^[28,29]^ In addition to these reported therapeutic effects, a large body of previous research has also pointed out that CBT interventions could be delivered through multiple modalities (e.g., internet-based interventions, individual in-person sessions, and group face-to-face meetings),^[30–32]^ which have all proved efficacious in alleviating a range of symptoms in patients with CFS, such as fatigue and functional impairment, further underscoring the potential of CBT.

However, as an emerging psychotherapeutic intervention, concerns regarding the efficacy and even safety of CBT remain widespread in the relevant research literature.^[33,34]^ Therefore, compared with previous reviews, this study comprehensively assesses and explores the clinical efficacy of CBT for CFS patients by including high-quality randomized controlled trials (RCTs), clearly defining diagnostic criteria for CFS patients, incorporating follow-up data, and employing subgroup analyses to investigate long-term efficacy. This provides reliable evidence to support the comprehensive management of CFS.

## 2. Methods

This meta-analysis was conducted in accordance with the Preferred Reporting Items for Systematic Reviews and Meta-Analyses guidelines,^[35]^ and the protocol was registered in the International Prospective Register of Systematic Reviews (Registration No.: CRD42023473925).

### 2.1. Search strategy

Two investigators independently conducted comprehensive searches of PubMed, Embase, Web of Science, and Cochrane Library from the time of database construction to March 2025. The search strategy combined both Medical Subject Headings terms and free-text terms. Search terms included “cognitive behavior therapy,” “chronic fatigue syndrome” and “randomized controlled trial” together with other related terms. The search was restricted to articles published in English. The detailed search strategies are shown in File S1, Supplemental Digital Content 1.

### 2.2. Inclusion criteria

Following the global Participant, Intervention, Comparison, Outcome, and Study framework, the primary selection criteria were established as follows:

Population: participants were eligible if they fulfilled either the Oxford^[36]^ or Centers for Disease Control and Prevention Criteria^[37]^ for CFS, with no restriction on age.

Intervention: CBT intervention or interventions based on CBT principles were eligible, with no restriction on CBT types and treatment durations, including face-to-face CBT, group CBT, and internet-based CBT (ICBT). Eligible interventions were those based on CBT principles, without restrictions on the types of CBT. To ensure a minimum threshold of therapeutic exposure, interventions were required to comprise at least 4 structured CBT sessions.

Comparison: the intervention forms of the comparison group included routine nursing, relaxation training, psychological education, or the waiting list.

Outcome: the primary outcome was fatigue score, and the secondary outcomes were physical function score, psychological outcomes, and adverse events.

Study design: RCTs.

### 2.3. Exclusion criteria

The following were exclusion criteria: single case study, revision, narrative review, or systematic review; non-RCTs research; duplicate studies; not accessible in full text; studies lacking complete data that could not be obtained; non-English language.

### 2.4. Study selection

Two reviewers independently evaluated articles by title and abstract to identify potentially relevant studies. Articles failing to satisfy the criteria for inclusion were excluded. The full-text articles of potentially relevant trials were then retrieved and independently evaluated by the 2 reviewers. The reasons for excluding studies during the full-text phase were documented. Disparities were discussed and resolved between the 2 reviewers, and when necessary, the third reviewer was consulted.

### 2.5. Data extraction

Data extraction included basic study information (study title, first author, country, date of publication), study design, interventions, control measures, study participants (number of individuals in intervention and control groups, mean age, gender), duration of intervention, follow-up period, and outcome indicators.

### 2.6. Risk of bias assessment

The quality of the included studies was evaluated using Cochrane risk-of-bias tool for randomized trials (RoB 2; Cochrane, London, UK).^[38]^ This tool can be used to assess bias across 5 domains: the randomization process, the intended intervention, missing outcome data, outcome measurements, and selection of reported results. According to RoB 2 guidelines, each study can be classified into one of 3 categories: low risk of bias (low risk across all domains), some concerns (concerns in at least 1 domain without high risk in any), or high risk of bias (high risk in at least 1 domain or some concerns in multiple domains raising substantial concerns about the results). Data extraction and quality assessment were independently performed by 2 reviewers. We involved a third reviewer if a consensus could not be reached. The agreement rate between the reviewers was calculated using kappa statistics.

### 2.7. Data analysis

The meta-analysis was performed using Review Manager (RevMan; The Cochrane Collaboration, London, UK) Software (version 5.4). For continuous variables, standardized mean difference (SMD) with 95% confidence intervals (CIs) was calculated as effect size measures. For dichotomous variables, such as adverse events, odds ratio with 95% CIs were computed. Heterogeneity among the included studies was evaluated using the *Q* test and *I*^2^ test, whereby an *I*^2^ value exceeding 50% indicated substantial heterogeneity, 25 to 50% indicated moderate heterogeneity, and below 25% implied insignificant heterogeneity. If *P* ≥ .1 and *I*^2^ ≤ 50%, a fixed-effect model was used for meta-analysis, and when *P* < .1 and *I*^2^ > 50%, a random-effect model was selected for meta-analysis. When significant heterogeneity was identified, we investigated potential sources using subgroup analysis, or sensitivity analysis was conducted by sequentially excluding each study to evaluate the robustness of the results. Publication bias was assessed by constructing a funnel plot in RevMan 5.4 and was statistically evaluated using Egger test in Stata 15.0. If the Egger test indicated significant publication bias, the trim-and-fill method was applied to adjust for its potential impact.

## 3. Results

### 3.1. Selection outcomes

A total of 1511 records were initially identified across 4 English databases. After removing 432 duplicates, 1079 records remained. Based on the predefined inclusion and exclusion criteria, 483 records were excluded for category mismatch. Subsequently, 596 records were screened by title and abstract, leaving 28 studies for full-text assessment. Of the 28 studies identified, 13 were excluded on the basis of the following criteria: hybrid study design (n = 2), absence of a control group (n = 3), intervention not based on CBT (n = 6), and data not being extractable (n = 2). Finally, 15 studies met the inclusion criteria and were included in the meta-analysis. The selection process is outlined in the Preferred Reporting Items for Systematic Reviews and Meta-Analyses flow diagram (Fig. 1).

**Figure 1.**
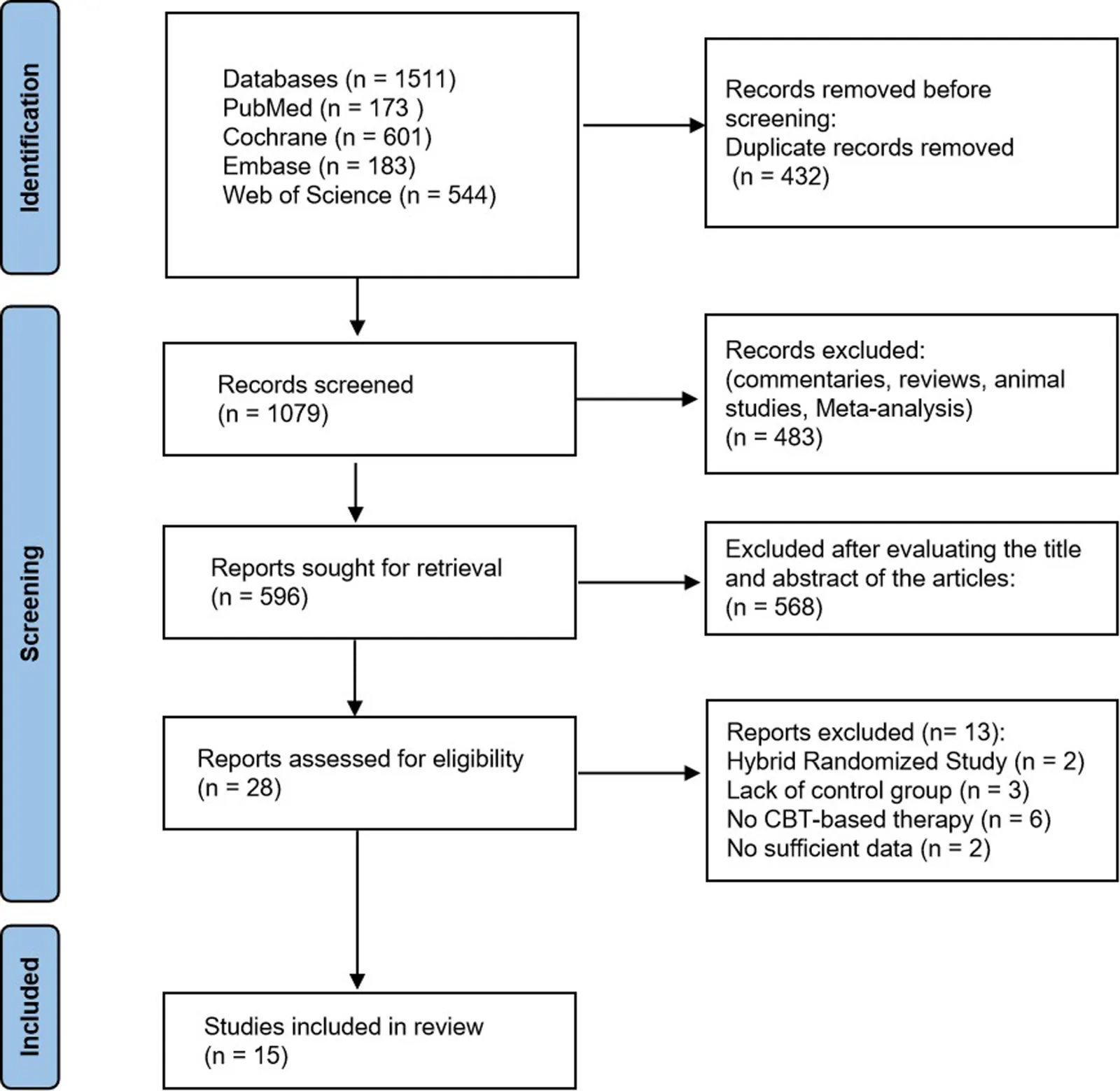
PRISMA flow diagram. PRISMA = Preferred Reporting Items for Systematic Reviews and Meta-Analyses.

### 3.2. Study characteristics

In total, 15 included studies involving 2270 participants were included,^[29,39–52]^ with sample sizes ranging from 23 to 161 across studies. All studies applied CBT-based interventions as stand-alone treatment, of which 10 studies employed individual CBT,^[29,40,42,43,46–51]^ 2 employed group CBT,^[45,52]^ and 3 employed ICBT.^[39,41,44]^

In the control group, 4 studies^[29,41,49,50]^ used waiting lists, 7 studies^[39,44–48,51]^ provided usual care, 3 applied psychoeducation,^[40,43,52]^ and 1 study^[42]^ used relaxation training. The duration of interventions ranged from 4 to 6 months, with a total of 8 to 16 sessions, and follow-up periods ranged from 3 to 24 months.

Primary outcomes included posttreatment or follow-up fatigue-related scales, such as the CFS, Checklist Individual Strength (CIS), and physical function subscale of the Short Form Health Survey-36 (SF-36 PF). In addition, the Symptom Checklist (SCL)-90 and Hospital Anxiety and Depression Scale (HADS) were used to evaluate changes in psychological health. Adverse events were explicitly reported in six studies. A summary of study characteristics is shown in Table 1.

**Table 1:** Characteristics of the studies included in the meta-analysis.

Study	Country	Mean age		Sample		Intervention		Outcomes	Duration of CBT	Measurement timepoint	Follow-up time	posttreartment Drop rate
		Experiment group	Control group	Experiment group	Control group	Experiment group	Control group					
Sharpe 1996	UK	34 ± 9.1	38 ± 11.8	30	30	16 CBT 4 M 1h Once weekly	Usual care	HADS Advent events	4 M		8 M	No reported
Prins 2001	Netherlands	36.2 ± 9.4	36.7 ± 10.3	93	91	16 CBT 8 M 1h	Usual care no treatment	CIS	8 M	8 M	14 M	10.8% (CBT) 14.3% (Con)
Stulemeijer 2005	Netherlands	15.6 ± 1.3	15.7 ± 1.3	36	35	10 CBT 5M	Waiting list	SF-36 PF	5 M	5 M		19.4% (CBT) 5.7% (Con)
O’Dowd 2006	UK	41.6	42.9	52	51	8 GCBT 4M 2h Once every 2 weeks	Usual care	CFQ SF-36 PF HADS	4 M	6M	6 M	No reported
Jason 2007	USA	43.8		29	28	13 CBT6 M 45min Once every 2 weeks	Relaxation 6 M 45min Once every 2 weeks	SF-36 PF	6 M	6M		20.7% (CBT) 10.7% (Con)
Chalder 2010	UK	15.0 ± 1.5	15.0 ± 3.0	32	31	13 CBT 6M1h Once every 2 weeks	Psychoeducation 6M 4 sessions	CFQ SF-36 PF Advent events	6 M	6 M	12 M	3.1% (CBT) 19.4% (Con)
White 2011	UK	39 ± 11	37 ± 11	161	160	14 CBT 6M	Usual care 12M 3 sessions	CFQ SF-36 PF HADS Advent events	6 M	6 M	13 M	9.3% (CBT) 5.0% (Con)
Nijhof 2012	Netherlands	15.9 ± 1.3	15.8 ± 1.3	68	67	ICBT 6M Once weekly	Usual care	CIS Advent events	6 M	6 M		1.5% (ICBT) 4.7% (Con)
Lloyd 2012	UK	15 ± 1.47	15 ± 2	32	31	13 CBT 6 M 1h	Psychoeducation 1h 3 sessions	CFQ SF-36 PF	6 M		24 M	25.0% (CBT) 35.5% (Con)
Tummers 2012	Netherlands	36.3 ± 12.1	36.4 ± 13.6	62	61	CBT 6M Once every 2 weeks	Waiting list	CIS	6M			11.3% (CBT) 8.2% (Con)
Sharpe 2015	UK	38.6 ± 12.0		161	160	14 CBT 6 M 2h	Usual care	CFQ SF-36 PF	6 M	6 M	24 M	27.3% (CBT) 28.1% (Con)
Wiborg 2015	Netherlands	36.4 ± 11.6 (CBTa) 39.9 ± 11. 3 (CBTb)	37.3 ± 10.8	68 68	68	14GCBT 6M 2h	Psychoeducation	SF-36 PF CIS SCL-90	6 M	6 M		14.7% (CBTa) 23.5% (CBTb) 11.8% (Con)
Janse 2018	Netherlands	36.6 ± 12.8 (CBTa) 36.4 ± 12.4 (CBTb)	39.9 ± 12.9	80 80	80	12 ICBT 6M Once every 2 weeks	Waiting list	CIS SF-36 PF SCL-90 Advent events	6 M	6 M		1.3% (CBTa) 1.3% (CBTb) 5.0% (Con)
Anderson 2020	UK	34 ± 9.1	38 ± 11.8	44	45	16 ICBT 6M	Usual care	Advent events	6 M	6 M		31.8% (ICBT) 33.3% (Con)
Gotaas 2021	Norway	37 ± 11 (CBT) 37 ± 11 (ICBT)	32 ± 10	78 78	78	CBT 4MOnce weekly ICBT 8M Once every 2 weeks	Waiting list	SF-36 PF CFQ Advent events	4 M	4 M		20.5% (CBT) 6.4% (ICBT) 10.0% (Con)

CBT = cognitive behavioral therapy, CFQ = Chalder Fatigue Questionnaire, CIS = Checklist Individual Strength, GCBT = group cognitive behavioral therapy, HADS = Hospital Anxiety and Depression Scale, ICBT = internet-based cognitive behavioral therapy, SCL-90 = Symptom Checklist 90, SF-36 PF = Physical function subscale of the Short Form Health Survey-36, UK = United Kingdom, USA = United States of America.

### 3.3. Assessment of the risk of bias

Overall, among the 15 included studies, 7^[29,40,41,45,46,48,50]^ were judged to have a low risk of bias, 6^[39,43,44,47,49,51]^ were judged to have some concerns, and 2^[42,52]^ were judged to have a high risk of bias (Fig. 2 and 3). More specifically, all included RCTs were assessed as having a low risk of bias in the randomization process (D1). Regarding deviations from the intended interventions (D2), 2 studies^[42,52]^ were rated as high risk due to a lack of blinding of participants and personnel, while 6 studies^[39,43,44,47,49,51]^ raised some concerns primarily because allocation concealment was not clearly reported. 7 studies^[29,40,41,45,46,48,50]^ implemented adequate blinding and were rated low risk in this domain. For missing outcome data (D3), all 15 studies were rated low risk, as outcome data were available for all or nearly all participants, and dropouts were reported in detail. In the measurement of the outcome (D4), all studies were rated low risk, as the outcome measures were unlikely to be influenced by knowledge of the intervention received. In the selection of the reported result (D5), all studies were rated low risk, with no evidence of selective reporting identified.

**Figure 2.**
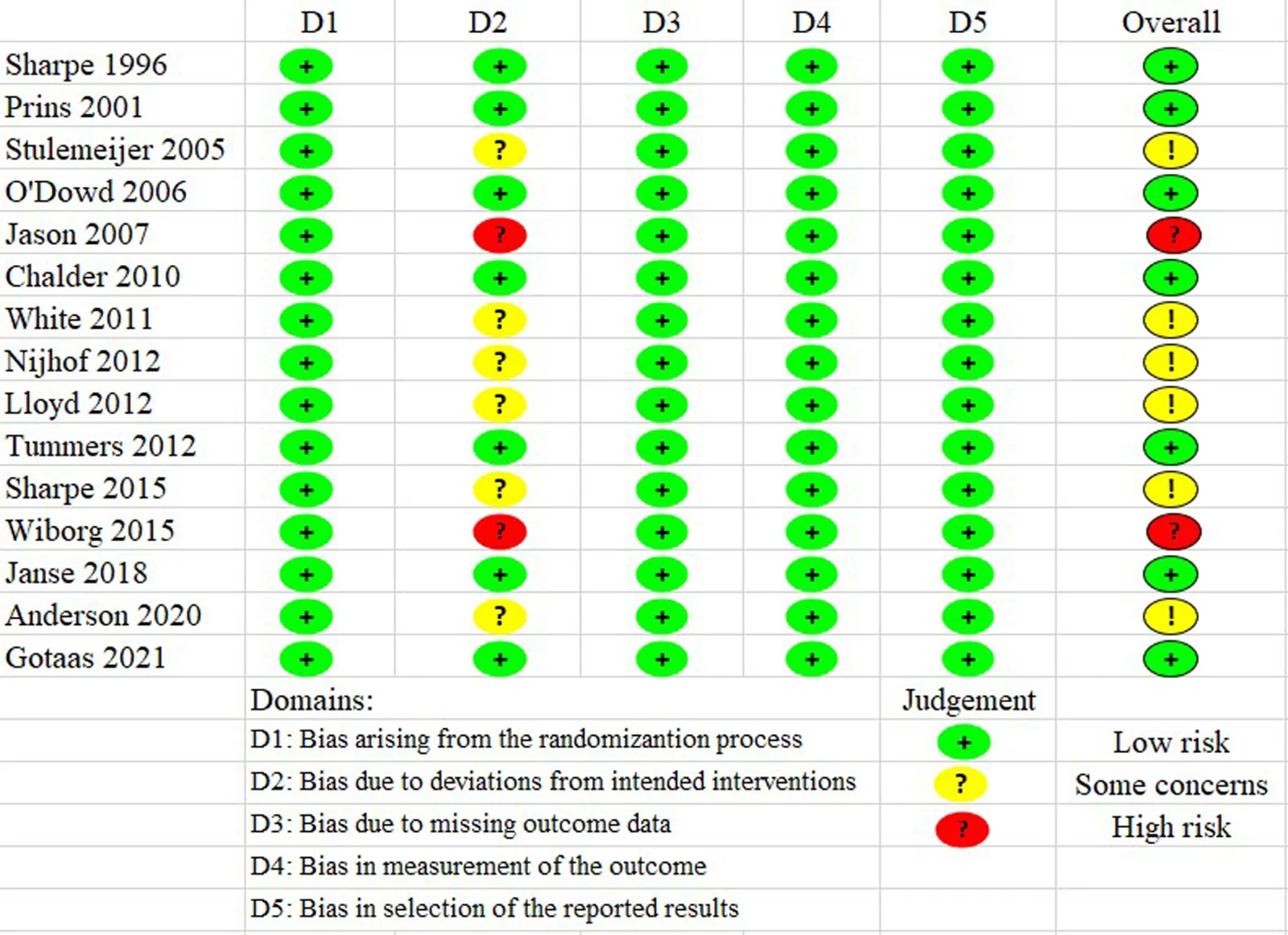
Risk of bias summary: review author’s judgements about each risk of bias item for each included study.

**Figure 3.**
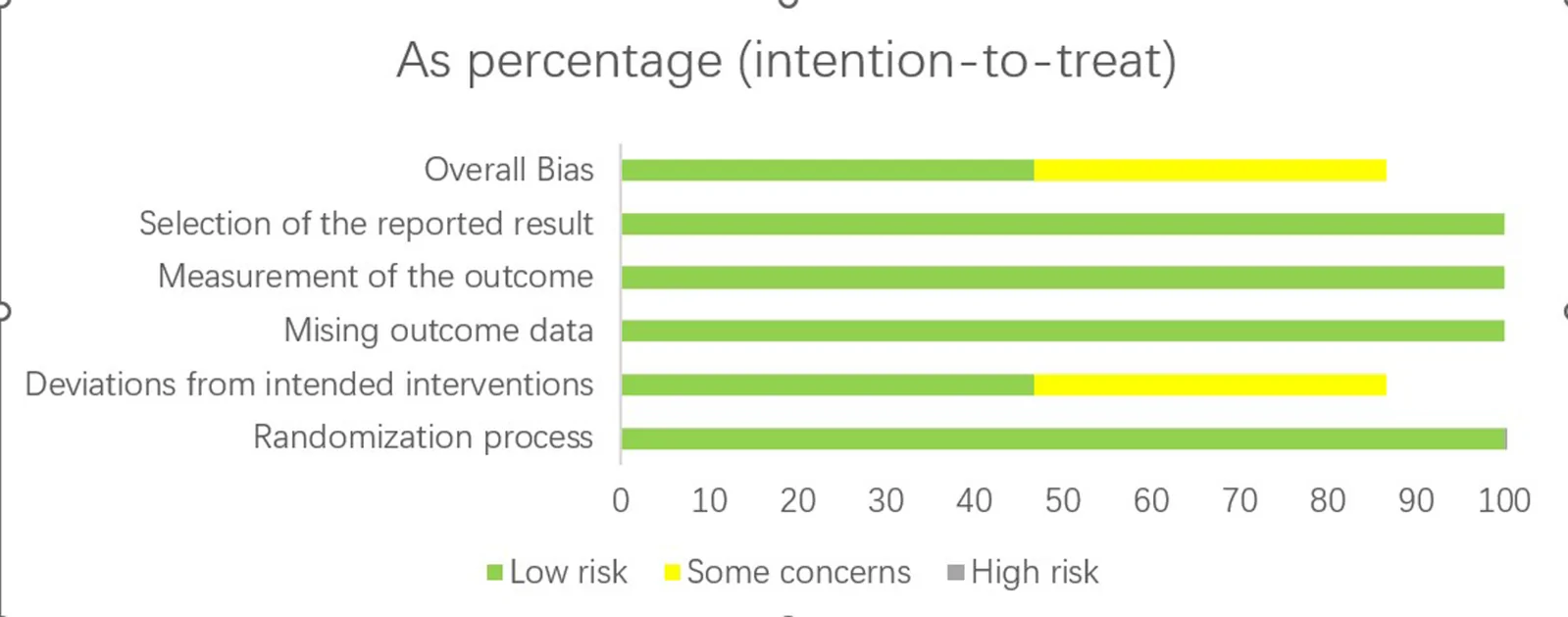
Risk of bias graph: review authors judgments about each risk of bias item presented as percentages across all included studies.

### 3.4. Meta-analysis

Prior to evaluating the effects of CBT, we assessed statistical heterogeneity across the included studies. Heterogeneity was low for most outcomes, including SF-36 PF (*I*^2^ = 17%, *P* = .27), Chalder Fatigue Questionnaire (CFQ) (*I*^2^ = 20%, *P* = .28), SCL-90 (*I*^2^ = 0%, *P* = .63), and adverse events (*I*^2^ = 0%, *P* = .66), and a fixed-effects model was accordingly employed. In contrast, the CIS outcome exhibited substantial heterogeneity (*I*^2^ = 76%, *P* = .001), which required a random-effects model. Detailed results for each outcome are presented below.

#### 3.4.1. SF-36 PF

A total of 10 studies involving 1828 patients with CFS and 13 comparisons were included to assess changes in physical function, and a fixed-effects model was employed. The results showed that CBT significantly improved physical function compared with the control group [SMD = 0.32, 95% CI (0.23–0.41), *P* < .01] (Fig. 4).

**Figure 4.**
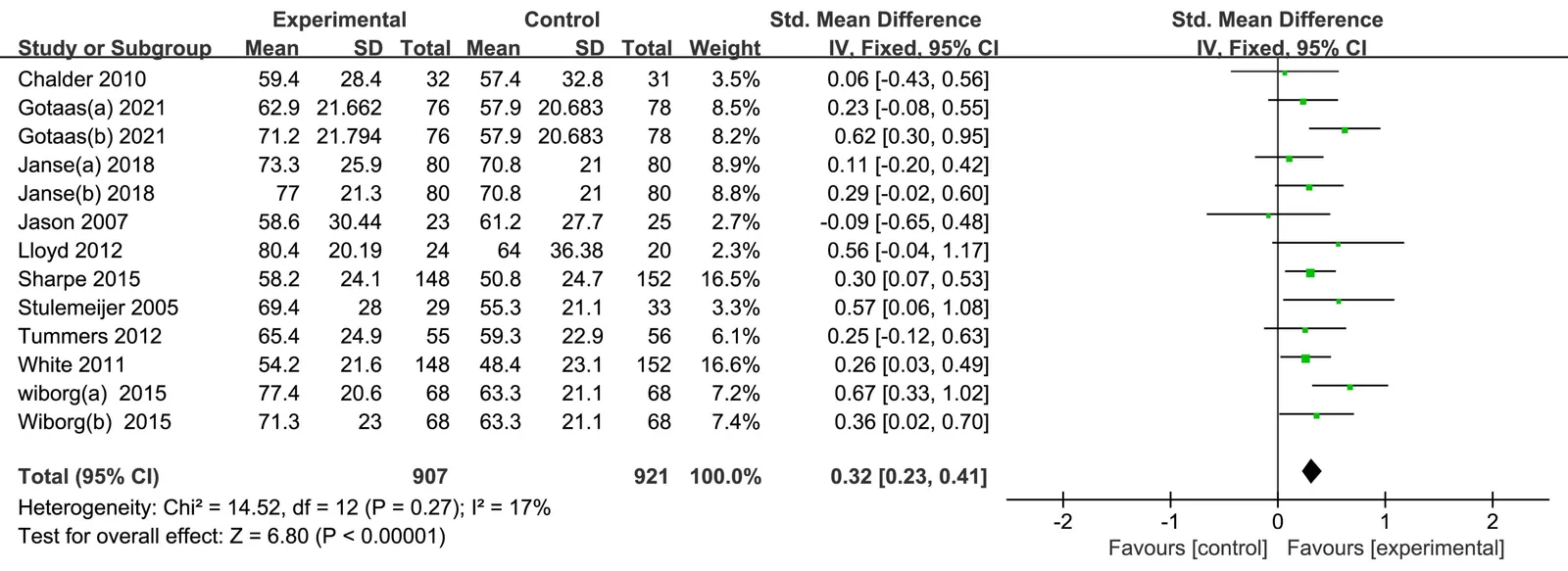
Forest plot of SF-36 PF. SF-36 PF = Physical function subscale of the Short Form Health Survey-36.

#### 3.4.2. CFQ

A total of 5 studies involving 1028 patients and 6 comparisons were included to assess changes in fatigue, and a fixed-effects model was used. The results supported that the CFQ score in the CBT group was significantly lower than that in the control group [SMD = −0.44, 95% CI (−0.57–−0.32), *P* < .01], suggesting that CBT is effective in alleviating fatigue in patients with CFS (Fig. 5).

**Figure 5.**
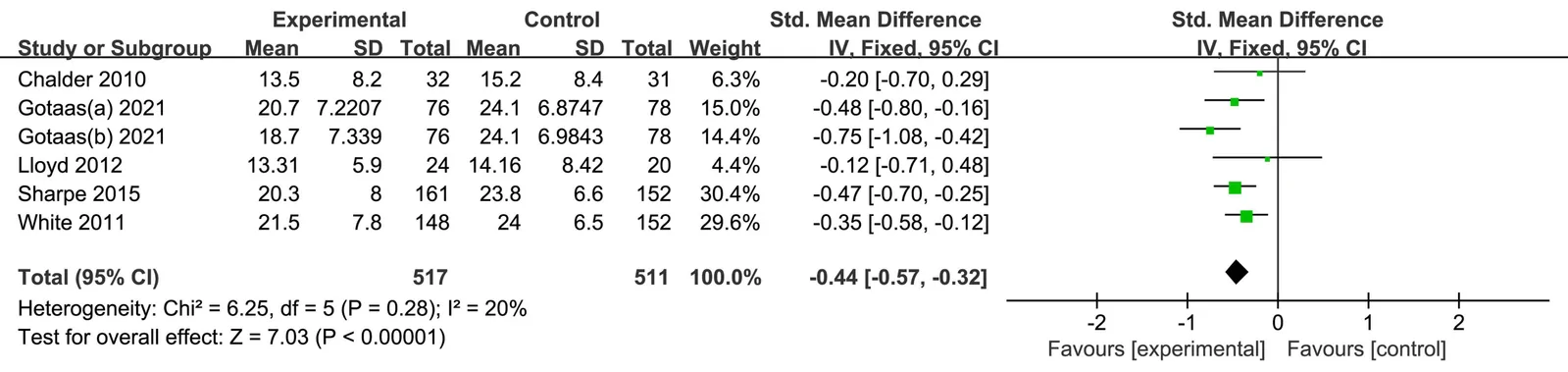
Forest plot of CFQ. CFQ = Chalder Fatigue Questionnaire.

#### 3.4.3. CIS

A total of 4 studies involving 908 patients and 6 comparisons were included to assess fatigue, and a random-effects model was used. The results showed that CBT significantly reduced fatigue compared with the control group [SMD = −0.88, 95% CI (−1.16–−0.60), *P* < .01] (Fig. 6).

**Figure 6.**
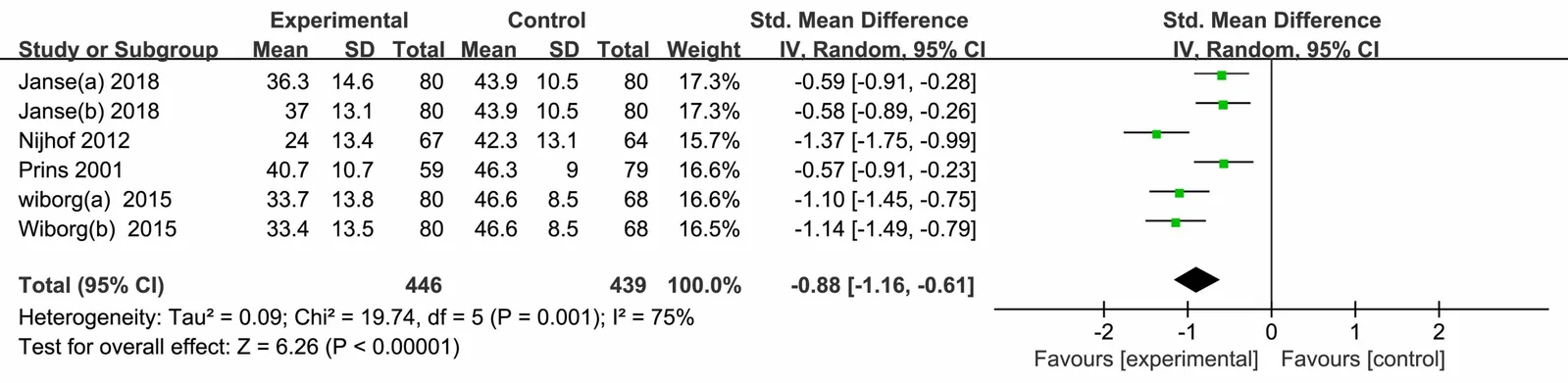
Forest plot of CIS. CIS = Checklist Individual Strength.

A sensitivity analysis was conducted using a leave-one-out method. Heterogeneity decreased to 67% only upon removal of the study by Nijhof et al,^[44]^ indicating that no single study substantially influenced the overall heterogeneity. Therefore, the results for the CIS outcomes are considered reliable, and the observed differences are likely attributable to factors unrelated to the intervention.

#### 3.4.4. SCL-90

A total of 2 studies with 489 participants and 3 outcome indicators were included, and a fixed-effects model was used. The results showed that CBT statistically improved psychological health compared with the control group [SMD = −0.44, 95% CI (−0.62–−0.26), *P* < .01] (Fig. 7).

**Figure 7.**
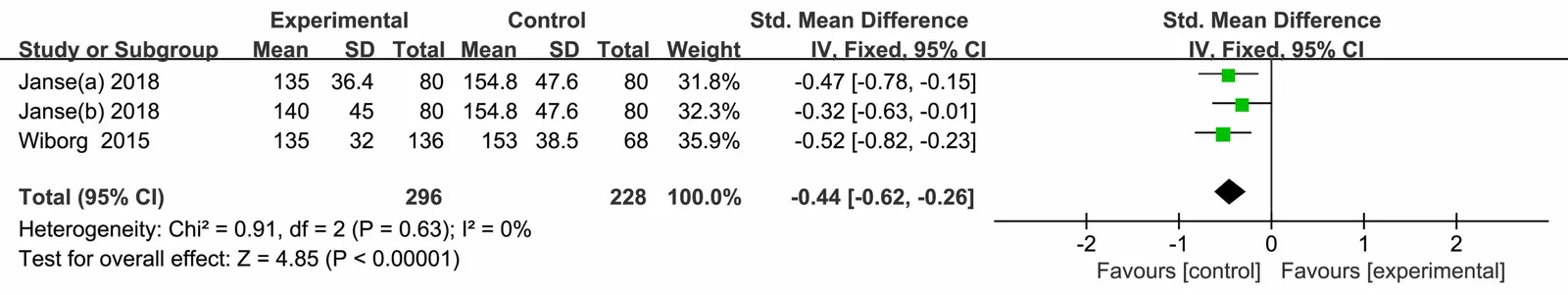
Forest plot of SCL-90. SCL = Symptom Checklist.

#### 3.4.5. Adverse events

A total of 5 studies involving 1085 patients and 7 comparisons were included to assess adverse events, and a fixed-effects model was applied. The results revealed no statistically significant difference in the incidence of adverse events between the 2 groups [odds ratio = 0.61, 95% CI (0.36–1.01), *P* = .06] (Fig. 8).

**Figure 8.**
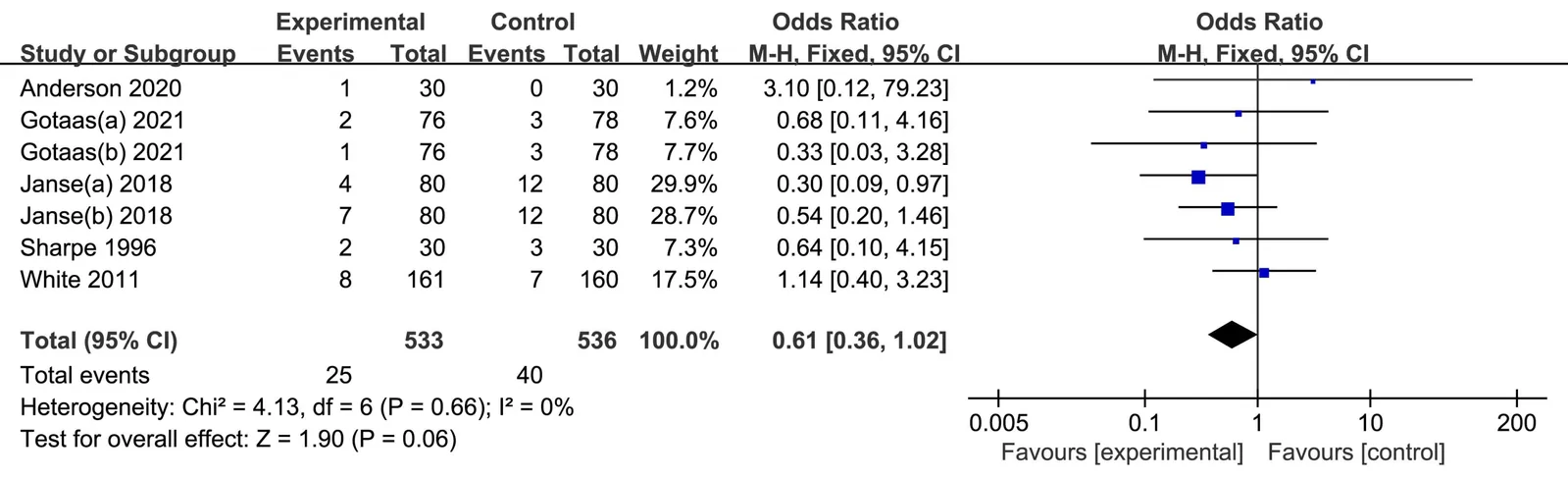
Forest plot of adverse events.

### 3.5. Subgroup analysis

#### 3.5.1. CFQ follow-up

A total of 6 studies involving 813 patients with CFS were included in this subgroup analysis to compare the effects of CBT and control interventions over different follow-up periods. The follow-up was categorized as short-term (≤ 6 months, n = 3) and long-term (6–24 months, n = 3). Heterogeneity was low in both the short-term (*I*^2^ = 0%, *P* = .44) and long-term follow-up groups (*I*^2^ = 0%, *P* = .49).

Analysis of the follow-up effects on fatigue revealed that short-term follow-up (< 6 months) was associated with a significant reduction in CFQ scores (SMD = −0.29, 95% CI: [−0.55–−0.03], *P* = .03), while long-term follow-up (> 6 months) also demonstrated sustained improvement (SMD = −0.35, 95% CI: [−0.50–−0.19], *P* < .01) (Fig. 8). Across both follow-up periods, heterogeneity remained low (*I*^2^ = 0%). Full details of the subgroup analysis are presented in Figure 9.

**Figure 9.**
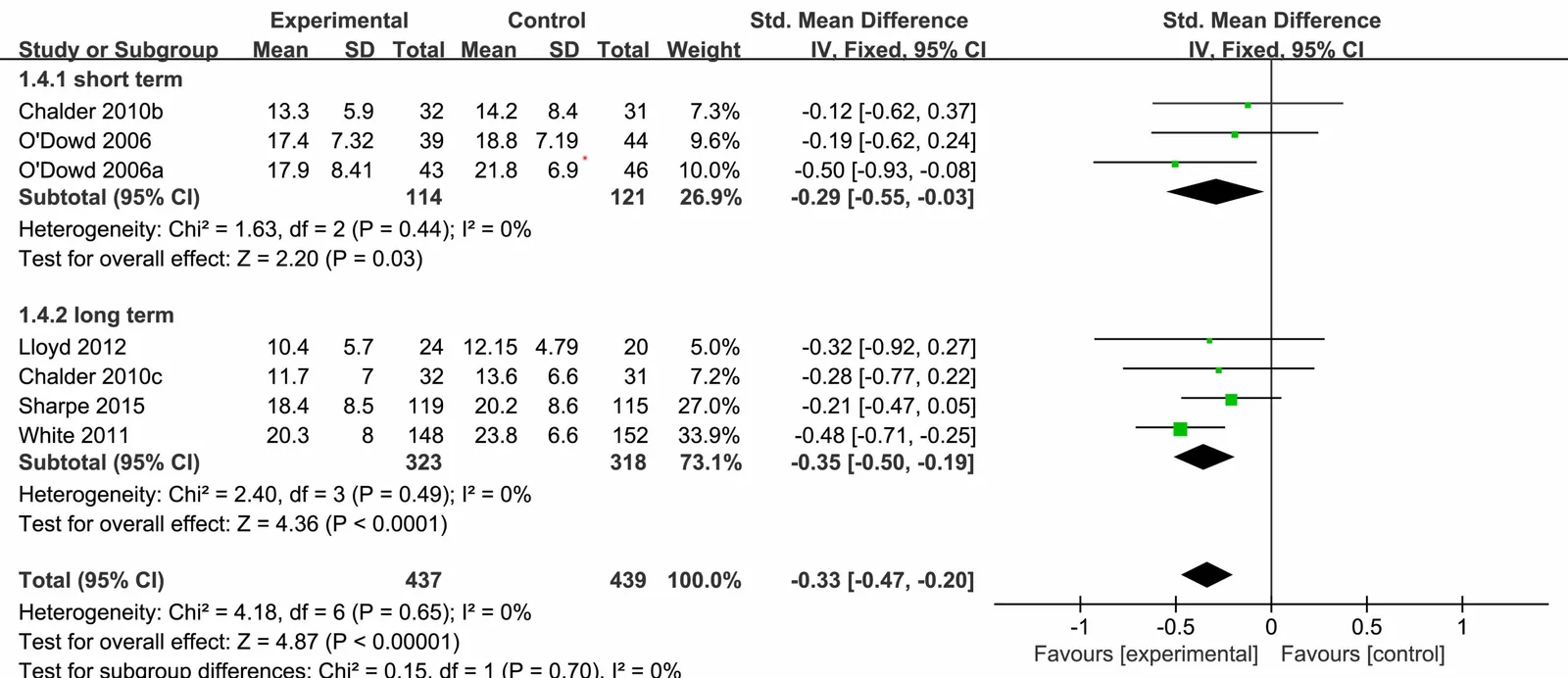
Subgroup analysis forest plot of CFQ follow-up. CFQ = Chalder Fatigue Questionnaire.

#### 3.5.2. SF-36 PF follow-up

This subgroup analysis included 5 studies involving 880 patients with CFS. The follow-up periods were categorized as short-term (≤ 6 months, n = 3) and long-term (6–24 months, n = 4). Heterogeneity was moderate in the short-term follow-up group (*I*^2^ = 32%, *P* = .23) and low in the long-term follow-up group (*I*^2^ = 0%, *P* = .69).

Further analysis showed that short-term follow-up was associated with a significant improvement in physical functioning (SMD = 0.31, 95% CI: [0.03–0.58], *P* = .03), while long-term follow-up also demonstrated sustained benefits (SMD = 0.21, 95% CI: [0.06–0.37], *P* = .006). Heterogeneity across studies remained low (*I*^2^ = 0%). Full details of the subgroup analysis are presented in Figure 10.

**Figure 10.**
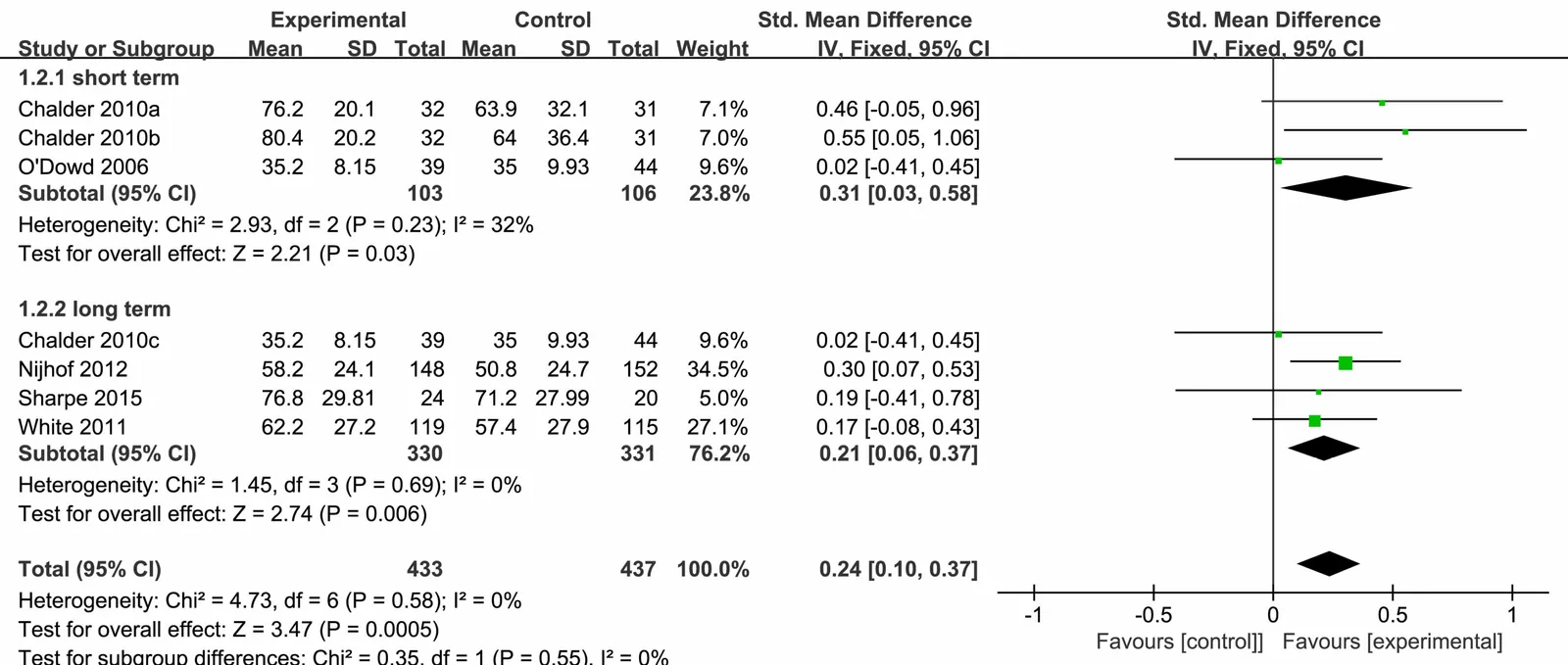
Subgroup analysis forest plot of SF-36 PF follow-up. SF-36 PF = Physical function subscale of the Short Form Health Survey-36.

#### 3.5.3. HADS follow-up

This subgroup analysis included 3 studies involving 928 patients with CFS. The assessments were divided into the HADS Depression scale (n = 3) and the HADS Anxiety scale (n = 3). Heterogeneity was low for both the HADS Depression scale (*I*^2^ = 12%, *P* = .32) and the HADS Anxiety scale (*I*^2^ = 0%, *P* = .57).

Further analysis showed that CBT was associated with a significant reduction in anxiety symptoms in the HADS Anxiety scale (SMD = −0.27, 95% CI: [−0.45–−0.09], *P* = .004) and a significant improvement in depression symptoms on the HADS Depression scale (SMD = −0.28, 95% CI: [−0.46–−0.10], *P* = .003), as shown in Figure 10. Heterogeneity across individual studies remained low (I^2^ = 0%). Full details of the subgroup analysis are presented in Figure 11.

**Figure 11.**
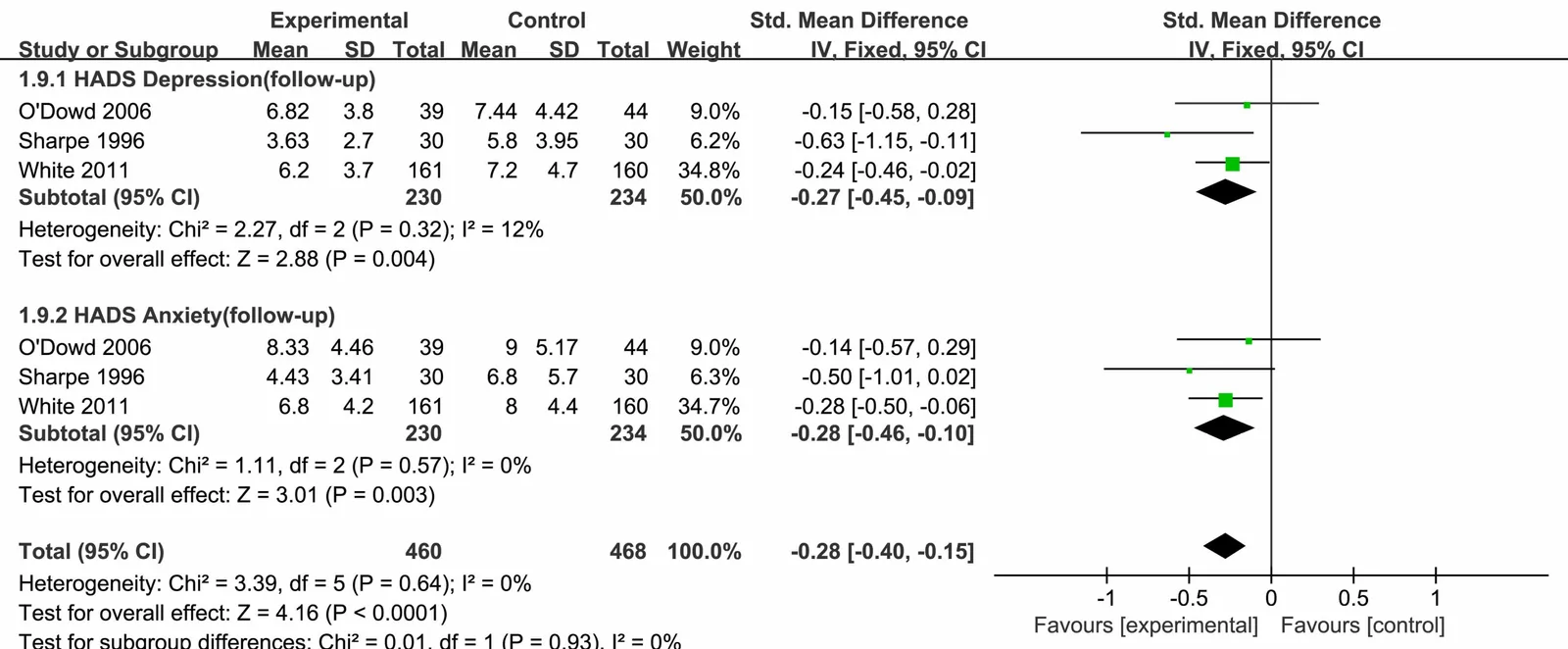
Subgroup analysis forest plot of HADS follow-up. HADS = Hospital Anxiety and Depression Scale.

### 3.6. Publication bias

The analysis of the SF-36 PF scale was used to assess publication bias, as it included 13 outcome parameters from 10 studies, covering a wide range of outcomes. Moreover, fewer than 10 studies have been conducted on other indicators. Publication bias was evaluated using visual inspection of funnel plots combined with Egger test, with *P* < .1 considered indicative of significant bias. The funnel plots showed no remarkable asymmetry, implying no obvious publication bias existed (Fig. 12). Egger test showed no statistically significant publication bias (*t* = 0.21, *P* = .834, *P* > .05), which was provided in Table 2.

**Table 2:** Results of Egger test for the impact of CBT for patients with CFS on SF-36 PF.

Std_Eff	Coef.	SE	*t*	*P* > t	95% CI	
Slope Bias	.3167851	.2229908	1.42	.181	−.1690701	.8026403
	.280078	1.311027	0.21	.834	−2.576405	3.136561

CBT = cognitive behavioral therapy, CFS = chronic fatigue syndrome, CI = confidence interval, SE = standard error, SF-36 PF = Physical function subscale of the Short Form Health Survey-36.

**Figure 12.**
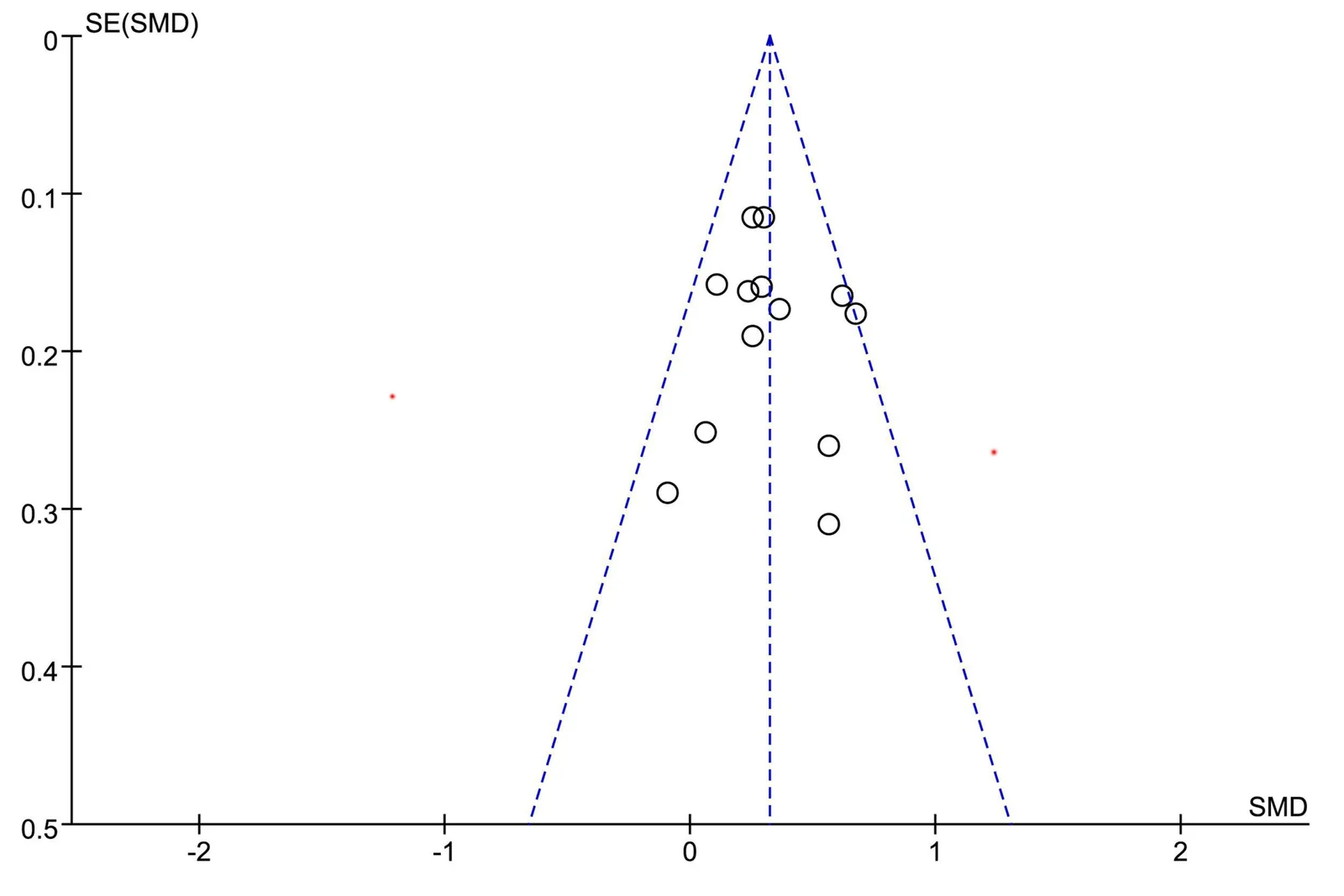
Funnel plot of the SF-36 PF. SF-36 PF = Physical function subscale of the Short Form Health Survey-36.

## 4. Discussion

This meta-analysis synthesized data from 15 RCTs, revealing that compared with control groups, the CBT intervention group demonstrated improvements in fatigue, physical functioning, and psychological health in both the short term and the long term. Furthermore, no significant difference in adverse events was observed between the groups. These findings suggest that CBT may have important clinical value for patients requiring pharmacological treatment or long-term rehabilitation, particularly those presenting with typical symptoms of CFS.

### 4.1. Effects of CBT for CFS on fatigue

Although the present meta-analysis confirms the significant short- and long-term efficacy of CBT for CFS fatigue, the literature reveals inconsistent findings, indicating that inconsistencies in treatment efficacy are objectively present. Firstly, discrepancies in intervention fidelity may arise because some studies within the broader literature did not fully implement or adequately train practitioners in the core principles of CBT for CFS.^[44,47,51]^ Differences in therapist competence and the overall effectiveness of implementation are also likely to influence observed outcomes. Secondly, heterogeneity among patient cohorts (including variations in disease severity, comorbidities, and the diagnostic criteria used for CFS) as well as differences in patient motivation and adherence, particularly in relation to baseline fatigue severity and co-occurring psychiatric symptoms, may affect individual responsiveness to CBT.^[53,54]^ Furthermore, the reliance on subjective rather than objective outcome measures, combined with the use of different comparison conditions (e.g., waiting list vs usual care), could contribute to the inconsistencies in reported treatment effects across studies.Numerous previous studies have examined the improvement in fatigue brought about by CBT through the interaction of the fear-avoidance model and the cognitive behavioral model.^[55–57]^ Specifically, due to cognitive deficits, patients may develop symptom misattribution, pain catastrophizing, and fear of activity.^[5]^ These negative cognitions can further lead to the avoidance of active rehabilitation behaviors, which in turn reinforce negative emotions, thus creating a vicious cycle that eventually results in long-term fatigue.^[58]^ Through cognitive restructuring, CBT may help patients to correct catastrophic interpretations of symptoms,^[50]^ dysfunctional assumptions,^[43]^ and misconceptions about their ability to engage in physical activity,^[59]^ thereby reducing activity-related fear and progressively interrupting the long-term maintenance of fatigue symptoms.

Based on the strong effect size and low heterogeneity observed for primary outcomes in this meta-analysis, a “strong” recommendation for incorporating CBT into CFS rehabilitation is recommended. Self-determination theory (SDT) posits that sustained behavior can only be achieved when an individual’s motivation is gradually internalized from external requirements into personal values and goals.^[60]^ Recent studies suggest that CBT approaches, such as Rational Emotive Behavior Therapy, can enhance self-determined motivation in physically inactive individuals, leading to improvements in both exercise behavior and motivational quality.^[61,62]^ Therefore, we propose integrating SDT with modularized CBT to reinforce cognitive behavioral processes in CFS patients. Under this framework, CBT should be structured according to SDT principles, with therapists delivering interventions that foster identity-based, integrative, and intrinsic forms of behavioral regulation.^[63]^ For example, early rehabilitative education may help patients understand and recognize the value of the rehabilitation plan in improving fatigue symptoms,^[64]^ guideline-based rehabilitation indicators can be internalized into individualized phased goals, and intervention modules can be flexibly combined according to each patient’s condition,^[65]^ thereby enhancing autonomy and persistence in the long-term rehabilitation process.

### 4.2. Effects of CBT for CFS on physical function

Consistent with a range of previous findings, the present study confirmed that CBT can improve the physical functioning of patients with CFS, indicating that CBT is feasible as a rehabilitative intervention in clinical practice.^[40,49,52]^ Moreover, this study revealed that the benefits of CBT for physical functioning are sustained over time, showing improvements both in the short term and long term. This conclusion is also supported by earlier studies.^[42,51]^ For example, Janse et al reported that improvements in fatigue and physical functioning following CBT could be maintained up to 12 months without symptom deterioration^[41]^.

The mechanisms underpinning CBT’s efficacy in CFS are multifaceted. While the fear-avoidance model provides a foundational framework, recent neurobiological evidence offers deeper insights. For instance, van der Schaaf et al used fMRI to demonstrate that CBT modulates resting-state connectivity between the sensorimotor network and the default mode network, potentially correlating with reduced fatigue and improved physical functioning.^[59,66]^This indicated that CBT’s benefits may extend beyond cognitive restructuring to include neuroplastic alterations. Future research should further investigate whether these changes in brain connectivity serve as mediators for CBT’s impact on clinical outcomes. Integrating psychological and neurobiological evidence in this way will be crucial for developing more targeted and effective CBT interventions.

Considering CBT’s broad functional benefits,^[67]^ its favorable short- and long-term effects,^[40]^ and applicability across different age groups,^[49,50]^ we strongly recommend CBT as a rehabilitation intervention to promote functional recovery in clinical practice. Previous studies have provided practical guidance for implementing CBT,^[47,52]^ including establishing baseline levels of activity and rest, promoting regular sleep patterns, and planning gradual increases in physical activity.^[27,68]^ In addition, when patients face setbacks during the recovery process, setting achievable rehabilitation goals and adopting graded activity therapy can help them gradually achieve comprehensive functional recovery and return to social life.^[69,70]^

### 4.3. Effects of CBT for CFS on mental health

Consistent with a range of earlier findings, this study further confirmed the effectiveness of CBT in improving the mental health of patients with CFS, including anxiety, depression, and other mental health aspects.^[49,50]^ Notably, prior research has indicated that mental distress is common among CFS patients,^[71]^ and that anxiety and depression can exacerbate fatigue, thereby limiting the effectiveness of interventions and hindering recovery.^[72,73]^ A recent study reported that reductions in anxiety often precede improvements in fatigue, whereas depression and fatigue appear to influence each other in both directions.^[67]^ CBT can help normalize anxiety and fatigue through cognitive and behavioral strategies, such as increasing understanding of CFS, managing energy levels, reducing excessive focus on symptoms, and enhancing self-efficacy.^[74,75]^ As a result, both mental health and fatigue are improved, contributing to better physical and psychological performance.

Furthermore, CBT has also demonstrated positive effects across various delivery formats.^[40]^ For instance, face-to-face CBT allows therapists to tailor interventions and respond quickly to symptom changes.^[29]^ Group-based CBT may provide extra benefits for patients with prominent depressive or anxiety symptoms by fostering peer support in a group setting.^[52]^ In addition, ICBT can increase accessibility and convenience by reducing commute time and other constraints.^[41]^ Taken together, these findings lead us to suggest that intervention formats should be selected according to each patient’s characteristics and circumstances, in order to optimize mental health and facilitate a quicker return to normal work and daily life.

### 4.4. Effects of CBT for CFS on safety

The results of this study showed that there was no significant difference in the incidence of adverse events between the CBT intervention group and the control group, supporting its use for the treatment of CFS, which is consistent with the conclusions of most previous studies. It should be noted that the efficacy of CBT may be affected by the levels of depression, anxiety, and somatic symptoms before treatment,^[67]^ suggesting that its efficacy has individual differences. For example, White et al observed adverse events in both control and intervention groups.^[51]^ Sharpe et al reported worsening depressive symptoms in the intervention group,^[48]^ which researchers attributed to baseline disease severity.

A differential response to CBT was also observed, with outcomes spanning from nonresponse to symptom deterioration in a proportion of patients. This highlights the critical influence of patient-level moderating factors. It is hypothesized that the presence of specific baseline characteristics (such as prominent fear-avoidance beliefs or cognitive fusion related to fatigue) identifies those most likely to benefit. In contrast, patients with severe PEM may benefit more from a CBT protocol tailored to energy conservation and activity pacing.

Due to inconsistent reporting, our meta-analysis could not precisely quantify the prevalence of these specific subtypes. In addition to individual differences among patients, some studies suggested CBT efficacy and safety may be influenced by therapist qualification, intervention frequency, and intervention duration.^[29,51]^ High-quality research is needed to clarify these factors. Consequently, CBT treatment requires comprehensive assessment of individual CFS patients to develop scientifically sound, tailored interventions.

### 4.5. Strengths and limitations

This meta-analysis integrated data from 15 RCTs involving 2270 participants. The use of a larger sample size (approximately 12.7% greater than that in the prior meta-analysis by Maas et al^[28]^) combined with rigorous methodology, enabled a more robust and reliable assessment, thereby advancing the evidence base for CBT in CFS. Our findings confirm that CBT confers significant physical and mental health benefits for CFS patients and quantitatively demonstrate that it does not increase adverse events relative to control conditions. A key methodological contribution of this study is its explicit analysis of long-term outcomes. By synthesizing follow-up data, we provide robust evidence for the sustained efficacy of CBT, addressing a critical gap in the existing literature. Furthermore, grounded in a comprehensive review of the literature, the study explores potential mechanisms through which CBT improves fatigue, physical functioning, and psychological health.

This study is subject to several limitations. First, the restriction to English-language RCTs may have introduced selection bias. Furthermore, the limited number of studies in certain subgroups precluded sensitivity analyses and a robust evaluation of moderating factors, such as specific CBT formats, and the absence of objective assessment methods may limit the generalizability of the findings. Third, despite employing random-effects models and subgroup analyses, the data remained inadequate for a definitive investigation of how baseline characteristics (e.g., fatigue and psychological symptom severity) influence outcomes. Future studies should include non-English publications, expand subgroup samples to enable precise moderator investigations, and stratify patients by baseline profiles to pinpoint optimal CBT modalities for personalized care.

## 5. Conclusion

In summary, this meta-analysis synthesizing evidence from 15 RCTs provides robust confirmation that CBT delivers significant and sustained benefits for patients with CFS. The findings demonstrate that CBT produces clinically meaningful improvements in core domains, including reduced fatigue severity, enhanced physical functioning, and alleviated psychological distress. Crucially, these positive effects are sustained during both short-term (≤6 months) and long-term (6–24 months) follow-up periods, highlighting the durability of CBT outcomes. Furthermore, CBT demonstrates good safety, with no significant increase in adverse event rates compared to control groups. These comprehensive findings strongly support the formal incorporation of CBT into standard clinical rehabilitation protocols for CFS. Its sustained efficacy warrants its prioritization as a core non-pharmacological intervention to facilitate wider adoption and implementation within healthcare systems.

However, a prudent evaluation grounded in precision medicine principles calls for a more nuanced interpretation. For more complex cases (such as those with severe symptoms or specific comorbidities) tailored approaches are necessary. Clinical practice should therefore incorporate individualized risk-benefit assessments and adjust intervention intensity accordingly, rather than assuming universal applicability. Future research should focus on optimizing CBT protocols for distinct patient subgroups and exploring synergistic effects with other therapies, including validating the combined efficacy of CBT and SDT through RCTs that target predefined comorbid subgroups with predetermined endpoints.

## Acknowledgments

We thank all the authors for their tireless assistance in this project. We sincerely appreciate the reviewers’ strict standards for quality and their valuable suggestions for further enhancements.

## Author contributions

**Conceptualization**: Qiang Huang.

**Data curation:** Junnan Liu, Qian Pei.

**Formal analysis:** Junnan Liu.

**Investigation:** Xiaoyu Jiang, Qian Pei.

**Methodology:** Junnan Liu, Qian Pei.

**Project administration:** Junnan Liu, Qian Pei.

**Supervision:** Xiaoyu Jiang.

**Validation:** Xiaoyu Jiang, Qian Pei.

**Visualization:** Xiaoyu Jiang.

**Writing – original draft:** Shulun Li, Xiaoyu Jiang.

**Writing – review & editing:** Shulun Li, Qiang Huang.



## References

[1] Bateman L Bested AC Bonilla HF. Myalgic encephalomyelitis/chronic fatigue syndrome: essentials of diagnosis and management. Mayo Clin Proc. 2021;96:2861–78. 10.1016/j.mayocp.2021.07.004 34454716

[2] Grach SL Seltzer J Chon TY Ganesh R. Diagnosis and management of myalgic encephalomyelitis/chronic fatigue syndrome. Mayo Clin Proc. 2023;98:1544–51. 10.1016/j.mayocp.2023.07.032 37793728

[3] Marshall-Gradisnik S Eaton-Fitch N. Understanding myalgic encephalomyelitis. Science. 2022;377:1150–1. 10.1126/science.abo1261 36074854

[4] Steiner S Fehrer A Hoheisel F. Understanding, diagnosing, and treating Myalgic encephalomyelitis/chronic fatigue syndrome - State of the art: report of the 2nd international meeting at the Charité Fatigue Center. Autoimmun Rev. 2023;22:103452. 10.1016/j.autrev.2023.103452 37742748

[5] Graves BS Patel M Newgent H. Chronic fatigue syndrome: diagnosis, treatment, and future direction. Cureus. 2024;16:e70616. 10.7759/cureus.70616 PMC11526618 39483544

[6] Nacul L O’Boyle S Palla L. How Myalgic Encephalomyelitis/Chronic Fatigue Syndrome (ME/CFS) Progresses: the natural history of ME/CFS. Front Neurol. 2020;11:826. 10.3389/fneur.2020.00826 PMC7431524 32849252

[7] Apostolou E Rosén A. Epigenetic reprograming in myalgic encephalomyelitis/chronic fatigue syndrome: a narrative of latent viruses. J Intern Med. 2024;296:93–115. 10.1111/joim.13792 38693641

[8] Arron HE Marsh BD Kell DB Khan MA Jaeger BR Pretorius E. Myalgic encephalomyelitis/chronic fatigue syndrome: the biology of a neglected disease. Front Immunol. 2024;15:1386607. 10.3389/fimmu.2024.1386607 PMC11180809 38887284

[9] Walitt B Singh K LaMunion SR. Deep phenotyping of post-infectious myalgic encephalomyelitis/chronic fatigue syndrome. Nat Commun. 2024;15:907. 10.1038/s41467-024-45107-3 PMC10881493 38383456

[10] Raanes EFW Stiles TC. Associations between psychological and immunological variables in chronic fatigue syndrome/myalgic encephalomyelitis: a systematic review. Front Psychiatry. 2021;12:716320. 10.3389/fpsyt.2021.716320 PMC8650213 34887782

[11] Van Campenhout J Buntinx Y Xiong HY. Unravelling the connection between energy metabolism and immune senescence/exhaustion in patients with myalgic encephalomyelitis/chronic fatigue syndrome. Biomolecules. 2025;15:357. 10.3390/biom15030357 PMC11940106 40149893

[12] Bjørklund G Dadar M Pivina L Doşa MD Semenova Y Maes M. Environmental, neuro-immune, and neuro-oxidative stress interactions in chronic fatigue syndrome. Mol Neurobiol. 2020;57:4598–607. 10.1007/s12035-020-01939-w 32761353

[13] Rometsch C Mansueto G Maas Genannt Bermpohl F Martin A Cosci F. Prevalence of functional disorders across Europe: a systematic review and meta-analysis. Eur J Epidemiol. 2024;39:571–86. 10.1007/s10654-024-01109-5 PMC11249491 38551715

[14] Lim EJ Ahn YC Jang ES Lee S-W Lee S-H Son C-G. Systematic review and meta-analysis of the prevalence of chronic fatigue syndrome/myalgic encephalomyelitis (CFS/ME). J Transl Med. 2020;18:100. 10.1186/s12967-020-02269-0 PMC7038594 32093722

[15] Wostyn P. COVID-19 and chronic fatigue syndrome: is the worst yet to come? Med Hypotheses. 2021;146:110469. 10.1016/j.mehy.2020.110469 PMC7836544 33401106

[16] Komaroff AL Lipkin WI. ME/CFS and Long COVID share similar symptoms and biological abnormalities: road map to the literature. Front Med (Lausanne). 2023;10:1187163. 10.3389/fmed.2023.1187163 PMC10278546 37342500

[17] Gilbert A. There is an overlap between long covid and ME/CFS. BMJ. 2023;383:2462. 10.1136/bmj.p2462 37875300

[18] Eaton-Fitch N Rudd P Er T Hool L Herrero L Marshall-Gradisnik S. Immune exhaustion in ME/CFS and long COVID. JCI Insight. 2024;9:e183810. 10.1172/jci.insight.183810 PMC11529985 39435656

[19] Chaves-Filho AM Braniff O Angelova A Deng Y Tremblay M-E. Chronic inflammation, neuroglial dysfunction, and plasmalogen deficiency as a new pathobiological hypothesis addressing the overlap between post-COVID-19 symptoms and myalgic encephalomyelitis/chronic fatigue syndrome. Brain Res Bull. 2023;201:110702. 10.1016/j.brainresbull.2023.110702 37423295

[20] Eckey M Li P Morrison B Bergquist J Davis RW Xiao W. Patient-reported treatment outcomes in ME/CFS and long COVID. Proc Natl Acad Sci U S A. 2025;122:e2426874122. 10.1073/pnas.2426874122 PMC12280984 40627388

[21] Komaroff AL Lipkin WI. Insights from myalgic encephalomyelitis/chronic fatigue syndrome may help unravel the pathogenesis of postacute COVID-19 syndrome. Trends Mol Med. 2021;27:895–906. 10.1016/j.molmed.2021.06.002 PMC8180841 34175230

[22] Blockmans D Persoons P. Long-term methylphenidate intake in chronic fatigue syndrome. Acta Clin Belg. 2016;71:407–14. 10.1080/17843286.2016.1200816 27351244

[23] Usón JRV Alecha MAI. Diagnostic and treatment challenges of chronic fatigue syndrome: role of immediate-release methylphenidate. Expert Rev Neurother. 2008;8:917–27. 10.1586/14737175.8.6.917 18505357

[24] Castro-Marrero J Segundo MJ Lacasa M Martinez-Martinez A Sentañes RS Alegre-Martin J. Effect of Dietary Coenzyme Q10 Plus NADH supplementation on fatigue perception and health-related quality of life in individuals with myalgic encephalomyelitis/chronic fatigue syndrome: a prospective, randomized, double-blind, placebo-controlled trial. Nutrients. 2021;13:2658. 10.3390/nu13082658 PMC8399248 34444817

[25] Mantle D Hargreaves IP Domingo JC Castro-Marrero J. Mitochondrial dysfunction and coenzyme Q10 supplementation in post-viral fatigue syndrome: an overview. Int J Mol Sci. 2024;25:574. 10.3390/ijms25010574 PMC10779395 38203745

[26] Henrich D Glombiewski JA Scholten S. Systematic review of training in cognitive-behavioral therapy: summarizing effects, costs and techniques. Clin Psychol Rev. 2023;101:102266. 10.1016/j.cpr.2023.102266 36963208

[27] Carson AJ McWhirter L. Cognitive behavioral therapy: principles, science, and patient selection in neurology. Semin Neurol. 2022;42:114–22. 10.1055/s-0042-1750851 35675820

[28] Bermpohl FMG Kucharczyk-Bodenburg AC Martin A. Efficacy and acceptance of cognitive behavioral therapy in adults with chronic fatigue syndrome: a meta-analysis. Int J Behav Med. 2024;31:895–910. 10.1007/s12529-023-10254-2 PMC11588766 38228869

[29] Gotaas ME Stiles TC Bjørngaard JH Borchgrevink PC Fors EA. Cognitive behavioral therapy improves physical function and fatigue in mild and moderate chronic fatigue syndrome: a consecutive randomized controlled trial of standard and short interventions. Front Psychiatry. 2021;12:580924. 10.3389/fpsyt.2021.580924 PMC8071989 33912079

[30] Kuut TA Müller F Csorba I. Efficacy of cognitive-behavioral therapy targeting severe fatigue following coronavirus disease 2019: results of a randomized controlled trial. Clin Infect Dis. 2023;77:687–95. 10.1093/cid/ciad257 PMC10495128 37155736

[31] May M Milrad SF Perdomo DM. Videoconference-delivered group cognitive behavioral stress management for ME/CFS patients who present with severe PEM: a randomized controlled trial. Fatigue. 2024;12:101–22. 10.1080/21641846.2024.2306801 PMC11086677 38736736

[32] Roshanghalb A Hansen B Rudoler D Best MW. Predicting CBT modality, treatment participation, and reliable improvements for individuals with anxiety and depression in a specialized mental health centre: a retrospective population-based cohort study. BMC Psychiatry. 2024;24:390. 10.1186/s12888-024-05817-w PMC11112857 38783227

[33] Roor JJ Dandachi-FitzGerald B Peters MJV Knoop H Ponds RWHM. Performance validity and outcome of cognitive behavior therapy in patients with chronic fatigue syndrome. J Int Neuropsychol Soc. 2022;28:473–82. 10.1017/S1355617721000643 34130768

[34] Heins MJ Knoop H Prins JB Stulemeijer M van der Meer JWM Bleijenberg G. Possible detrimental effects of cognitive behaviour therapy for chronic fatigue syndrome. Psychother Psychosom. 2010;79:249–56. 10.1159/000315130 20502065

[35] Moher D Liberati A Tetzlaff J Altman DG; PRISMA Group. Preferred reporting items for systematic reviews and meta-analyses: the PRISMA statement. PLoS Med. 2009;6:e1000097. PMC3090117 21603045

[36] Sharpe MC Archard LC Banatvala JE. A report--chronic fatigue syndrome: guidelines for research. J R Soc Med. 1991;84:118–21. 10.1177/014107689108400224 PMC1293107 1999813

[37] Fukuda K Straus SE Hickie I Sharpe MC Dobbins JG Komaroff A. The chronic fatigue syndrome: a comprehensive approach to its definition and study. International Chronic Fatigue Syndrome Study Group. Ann Intern Med. 1994;121:953–9. 10.7326/0003-4819-121-12-199412150-00009 7978722

[38] Sterne JAC Savović J Page MJ. RoB 2: a revised tool for assessing risk of bias in randomised trials. BMJ. 2019;366:l4898. 10.1136/bmj.l4898 31462531

[39] Anderson E Parslow R Hollingworth W. Recruiting adolescents with chronic fatigue syndrome/myalgic encephalomyelitis to internet-delivered therapy: internal pilot within a randomized controlled trial. J Med Internet Res. 2020;22:e17768. 10.2196/17768 PMC7450376 32784188

[40] Chalder T Deary V Husain K Walwyn R. Family-focused cognitive behaviour therapy versus psycho-education for chronic fatigue syndrome in 11- to 18-year-olds: a randomized controlled treatment trial. Psychol Med. 2010;40:1269–79. 10.1017/S003329170999153X 19891804

[41] Janse A Worm-Smeitink M Bleijenberg G Donders R Knoop H. Efficacy of web-based cognitive-behavioural therapy for chronic fatigue syndrome: randomised controlled trial. Br J Psychiatry. 2018;212:112–8. 10.1192/bjp.2017.22 29436329

[42] Jason LA Torres-Harding S Friedberg F. Non-pharmacological interventions for CFS: a randomized trial. J Clin Psychol Med Settings. 2007;14:275–96.

[43] Lloyd S Chalder T Rimes KA. Family-focused cognitive behaviour therapy versus psycho-education for adolescents with chronic fatigue syndrome: long-term follow-up of an RCT. Behav Res Ther. 2012;50:719–25. 10.1016/j.brat.2012.08.005 22985998

[44] Nijhof SL Bleijenberg G Uiterwaal CS Kimpen JLL van de Putte EM. Effectiveness of internet-based cognitive behavioural treatment for adolescents with chronic fatigue syndrome (FITNET): a randomised controlled trial. Lancet. 2012;379:1412–8. 10.1016/S0140-6736(12)60025-7 22385683

[45] O’Dowd H Gladwell P Rogers CA Hollinghurst S Gregory A. Cognitive behavioural therapy in chronic fatigue syndrome: a randomised controlled trial of an outpatient group programme. Health Technol Assess. 2006;10:iii–v, ix. 10.3310/hta10370 17014748

[46] Prins JB Bleijenberg G Bazelmans E. Cognitive behaviour therapy for chronic fatigue syndrome: a multicentre randomised controlled trial. Lancet. 2001;357:841–7. 10.1016/S0140-6736(00)04198-2 11265953

[47] Sharpe M Goldsmith KA Johnson AL Chalder T Walker J White PD. Rehabilitative treatments for chronic fatigue syndrome: long-term follow-up from the PACE trial. Lancet Psychiatry. 2015;2:1067–74. 10.1016/S2215-0366(15)00317-X 26521770

[48] Sharpe M Hawton K Simkin S. Cognitive behaviour therapy for the chronic fatigue syndrome: a randomized controlled trial. BMJ. 1996;312:22–6. 10.1136/bmj.312.7022.22 PMC2349693 8555852

[49] Stulemeijer M de Jong LW Fiselier TJ Hoogveld SWB Bleijenberg G. Cognitive behaviour therapy for adolescents with chronic fatigue syndrome: randomised controlled trial. BMJ. 2005;330:14. 10.1136/bmj.38301.587106.63 PMC539840 15585538

[50] Tummers M Knoop H van Dam A Bleijenberg G. Implementing a minimal intervention for chronic fatigue syndrome in a mental health centre: a randomized controlled trial. Psychol Med. 2012;42:2205–15. 10.1017/S0033291712000232 22354999

[51] White PD Goldsmith KA Johnson AL. Comparison of adaptive pacing therapy, cognitive behaviour therapy, graded exercise therapy, and specialist medical care for chronic fatigue syndrome (PACE): a randomised trial. Lancet. 2011;377:823–36. 10.1016/S0140-6736(11)60096-2 PMC3065633 21334061

[52] Wiborg JF van Bussel J van Dijk A Bleijenberg G Knoop H. Randomised controlled trial of cognitive behaviour therapy delivered in groups of patients with chronic fatigue syndrome. Psychother Psychosom. 2015;84:368–76. 10.1159/000438867 26402868

[53] Crawley E Anderson E Cochrane M. Comparison of cognitive behaviour therapy versus activity management, both delivered remotely, to treat paediatric chronic fatigue syndrome/myalgic encephalomyelitis: the UK FITNET-NHS RCT. Health Technol Assess. 2024;28:1–134. 10.3310/VLRW6701 PMC11590115 39485730

[54] Albers E Nijhof LN Berkelbach van der Sprenkel EE van de Putte EM Nijhof SL Knoop H. Effectiveness of internet-based cognitive behavior therapy (fatigue in teenagers on the internet) for adolescents with chronic fatigue syndrome in routine clinical care: observational study. J Med Internet Res. 2021;23:e24839. 10.2196/24839 PMC8398746 34397389

[55] Stahl D Rimes KA Chalder T. Mechanisms of change underlying the efficacy of cognitive behaviour therapy for chronic fatigue syndrome in a specialist clinic: a mediation analysis. Psychol Med. 2014;44:1331–44. 10.1017/S0033291713002006 23931831

[56] Bloot L Heins MJ Donders R Bleijenberg G Knoop H. The process of change in pain during cognitive-behavior therapy for chronic fatigue syndrome. Clin J Pain. 2015;31:914–21. 10.1097/AJP.0000000000000191 25503595

[57] Geraghty KJ Blease C. Myalgic encephalomyelitis/chronic fatigue syndrome and the biopsychosocial model: a review of patient harm and distress in the medical encounter. Disabil Rehabil. 2019;41:3092–102. 10.1080/09638288.2018.1481149 29929450

[58] Brooks SK Chalder T Rimes KA. Chronic fatigue syndrome: cognitive, behavioural and emotional processing vulnerability factors. Behav Cogn Psychother. 2017;45:156–69. 10.1017/S1352465816000631 28098051

[59] van der Schaaf ME Geerligs L Toni I Knoop H Oosterman JM. Disentangling pain and fatigue in chronic fatigue syndrome: a resting state connectivity study before and after cognitive behavioral therapy. Psychol Med. 2024;54:1735–48. 10.1017/S0033291723003690 38193344

[60] Huang H Wang HHX Donaghy E Henderson D Mercer SW. Using self-determination theory in research and evaluation in primary care. Health Expect. 2022;25:2700–8. 10.1111/hex.13620 PMC9700158 36181716

[61] Turner MJ Frost N Outar L. Rational Emotive Behavior Therapy for exercise: examining self-determined motivation, alongside readiness, confidence, and motivation to exercise. Front Psychol. 2025;16:1557885. 10.3389/fpsyg.2025.1557885 PMC12305818 40735175

[62] King AM Plateau CR Turner MJ Young P Barker JB. A systematic review of the nature and efficacy of Rational Emotive Behaviour Therapy interventions. PLoS One. 2024;19:e0306835. 10.1371/journal.pone.0306835 PMC11232995 38980891

[63] Ryan RM. Motivation, movement, and vitality: self-determination theory and its organismic perspective on physical activity as part of human flourishing. Psychol Sport Exerc. 2025;80:102932. 10.1016/j.psychsport.2025.102932 40623601

[64] Sharpe M Chalder T White PD. Evidence-based care for people with chronic fatigue syndrome and myalgic encephalomyelitis. J Gen Intern Med. 2022;37:449–52. 10.1007/s11606-021-07188-4 PMC8811058 34791590

[65] Ntoumanis N Moller AC. Self-determination theory informed research for promoting physical activity: contributions, debates, and future directions. Psychol Sport Exerc. 2025;80:102879. 10.1016/j.psychsport.2025.102879 40383282

[66] Lee JS Sato W Son CG. Brain-regional characteristics and neuroinflammation in ME/CFS patients from neuroimaging: a systematic review and meta-analysis. Autoimmun Rev. 2024;23:103484. 10.1016/j.autrev.2023.103484 38016575

[67] Van Oudenhove L Debyser S Vergaelen E Claes S Van Den Houte M. Predictors of treatment response trajectories to cognitive behavioral therapy for chronic fatigue syndrome: a cohort study. J Psychosom Res. 2024;187:111954. 10.1016/j.jpsychores.2024.111954 39423465

[68] Kazantzis N. Cognitive behavioral therapy: strategies for enhancing treatment engagement. Psychiatr Clin North Am. 2024;47:301–10. 10.1016/j.psc.2024.03.002 38724121

[69] Tolin DF Lord KA Knowles KA. Cognitive-behavioral therapy enhancement strategies. Psychiatr Clin North Am. 2024;47:355–65. 10.1016/j.psc.2024.02.005 38724125

[70] Norbury A Hauser TU Fleming SM Dolan RJ Huys QJM. Different components of cognitive-behavioral therapy affect specific cognitive mechanisms. Sci Adv. 2024;10:eadk3222. 10.1126/sciadv.adk3222 PMC10971416 38536924

[71] White P Abbey S Angus B. Anomalies in the review process and interpretation of the evidence in the NICE guideline for chronic fatigue syndrome and myalgic encephalomyelitis. J Neurol Neurosurg Psychiatry. 2023;94:1056–63. 10.1136/jnnp-2022-330463 37434321

[72] Wright A Fisher PL Baker N O’Rourke L Cherry MG. Perfectionism, depression and anxiety in chronic fatigue syndrome: a systematic review. J Psychosom Res. 2021;140:110322. 10.1016/j.jpsychores.2020.110322 33278659

[73] Stevelink SAM Mark KM Fear NT Hotopf M Chalder T. Chronic fatigue syndrome and occupational status: a retrospective longitudinal study. Occup Med (Lond). 2022;72:177–83. 10.1093/occmed/kqab170 34865116

[74] Jacobsen HB Glette M Hara KW Stiles TC. Metacognitive beliefs as predictors of return to work after intensive return-to-work rehabilitation in patients with chronic pain, chronic fatigue and common psychological disorders: results from a prospective trial. Front Psychol. 2020;11:70. 10.3389/fpsyg.2020.00070 PMC7025452 32116900

[75] Clark C Holttum S. A life I can cope with.” An alternative model of cognitive behavioural therapy (CBT) for CFS/ME. Health Expect. 2022;25:91–102. 10.1111/hex.13326 PMC8849251 34472690

