# Supplementary material for: Rehabilitative efficacy of cognitive behavioral therapy-based intervention for chronic fatigue syndrome: A systematic review and meta-analysis
Source: Medicine (Baltimore). 2026 Oct 9;105(41):e50765. doi: 10.1097/MD.0000000000050765 (PMC13657625; doi:10.1097/MD.0000000000050765)
Supplement: Supplementary file 1 [file medi-105-e50765-s001.docx]

**Search strategy**

**PubMed**

#1 “[Cognitive behavioral therapy](https://pubmed.ncbi.nlm.nih.gov/31599606/)”[Mesh] OR “[cognitive behavioral therapy](https://pubmed.ncbi.nlm.nih.gov/31599606/)”[Title/Abstract] OR “cognitive therapy”[Title/Abstract] OR “cognitive behavio*”[Title/Abstract] OR CBT [Title/Abstract]

#2 “Chronic fatigue syndrome”[MeSH term] OR “chronic fatigue syndrome” [Title/Abstract] OR CFS[Title/Abstract]

#3 “randomized controlled trial”[Mesh] OR “controlled clinical trials”[Title/Abstract] OR randomized [Title/Abstract] OR randomly[Title/Abstract] OR RCT[Title/Abstract]

#4 #1 AND #2 AND #3

**Web of Science**

#1 TS = (“Cognitive behavioral therapy”) OR TI = (“Cognitive behavioral therapy” or “cognitive therapy” OR “cognitive behavio*” OR “CBT”))

#2 TS = (“Chronic fatigue syndrome”) OR TI = (“Chronic fatigue syndrome” OR “Chronic Fatigue Disorder” OR “CFS”))

#3 TS = (“randomized controlled trial”) OR TI = (“controlled clinical trial” OR “randomized” OR “RCT”))

#4 #1 AND #2 AND #3

**Embase**

#1	'[Cognitive behavioral therapy](https://pubmed.ncbi.nlm.nih.gov/31599606/)'/exp OR '[cognitive behavioral therapy](https://pubmed.ncbi.nlm.nih.gov/31599606/)':ti,ab OR ' cognitive therapy':ti,ab OR 'cognitive behavio*':ti,ab OR 'CBT':ti,ab

#2	'[Chronic fatigue syndrome](https://pubmed.ncbi.nlm.nih.gov/31599606/)'/exp OR 'chronic fatigue syndrome':ti,ab OR 'CFS':ti,ab

#1	'randomized controlled trial'/exp OR ' controlled clinical trials':ti,ab OR 'randomized ':ti,ab OR 'RCT':ti,ab

#4 #1 AND #2 AND #3

**Cocharane**

#1 Mesh“[Cognitive behavioral therapy](https://pubmed.ncbi.nlm.nih.gov/31599606/)” OR “[cognitive behavioral therapy](https://pubmed.ncbi.nlm.nih.gov/31599606/)”:ti,ab,kw OR “cognitive therapy”:ti,ab,kw OR “cognitive behavio*”:ti,ab,kw OR “CBT”:ti,ab,kw

#2	Mesh“[Chronic fatigue syndrome](https://pubmed.ncbi.nlm.nih.gov/31599606/)” OR “chronic fatigue syndrome”:ti,ab,kw OR “CFS”:ti,ab,kw

#3	“randomized controlled trial”/exp OR “controlled clinical trials”:ti,ab,kw OR “randomized”:ti,ab,kw OR “RCT”:ti,ab,kw

#4 #1 AND #2 AND #3
